# Eye-blinks in choice response tasks uncover hidden aspects of information processing

**DOI:** 10.17179/excli2015-696

**Published:** 2015-11-27

**Authors:** Edmund Wascher, Holger Heppner, Tina Möckel, Sven Oliver Kobald, Stephan Getzmann

**Affiliations:** 1 IfADo - Leibniz Research Centre For Working Environment and Human Factors, Ardeystr. 67, 44139 Dortmund, Germany

**Keywords:** eye-blinks, EOG, cognitive control, attention, natural marker

## Abstract

Spontaneous eye-blinks occur much more often than it would be necessary to maintain the tear film on the eyes. Various factors like cognitive demand, task engagement, or fatigue are influencing spontaneous blink rate. During cognitive information processing there is evidence that blinks occur preferably at moments that can be assigned to input stream segmentation. We investigated blinking behavior in three different visual choice response experiments (Experiment 1: spatial Stimulus-Response correspondence, Experiment 2: Change Detection, Experiment 3: Continuous performance Test - AX version). Blinks during the experimental tasks were suppressed when new information was expected, as well as during cognitive processing until the response was executed. Blinks in go trials occurred within a short and relatively constant interval after manual responses. However, blinks were not a side effect of manual behavior, as they occurred in a similar manner in no-go trials in which no manual response was executed. In these trials, blinks were delayed when a prepared response had to be inhibited, compared to trials in which no response was intended. Additionally, time on task effects for no-go blinks mirrored those obtained in go trials. Thus, blinks seem to provide a reliable measure for cognitive processing beyond (or rather additional to) manual responses.

## Introduction

Every fifth second - on average - we are closing our eyes for a short moment without being aware of it. It does not seem to change anything regarding our representation of the outer world. But is this really the truth? The present study aims at the question what the seemingly trivial reflex of spontaneous eye blinking means to human information processing.

Three types of eye blinks are distinguished in the literature: Besides a) voluntary blinking and b) reflexive blinking (corneal reflex in order to protect the eye) which have a clear purpose each, c) spontaneous or endogenous blinking occurs frequently and mostly unaware several times per minute (Alm and Kaufman, 2002[1]; Cong et al., 2010[11]; Lawson, 1948[32]). The assumed purpose of endogenous blinks is the maintenance of the tear film on the cornea. However, the average blink rate is, with a huge inter- and intra-individual variance, around 14 blinks per minute (Doughty and Naase, 2006[13]). This is much higher than it would be necessary to preserve eye moisture (Evinger, 1995[16]; Sweeney et al., 2013[48]), because the “tear break-up time“ in healthy adults is normally above 10s and about 27s on average (Sweeney et al., 2013[48]).

One possible origin of variation in blink rates is the dopaminergic system. There is conclusive evidence that high dopaminergic activity increases and low dopaminergic activity decreases blink rate. While patients suffering from Parkinson's disease show reduced eye blink rates (Karson, 1983[27]), the amount of blinks tends to increase in schizophrenic patients (Karson et al., 1990[28]). Also, the application of dopamine agonists or antagonists changes eye blink rates in monkeys in the same direction (Jutkiewicz and Bergman, 2004[26]; Kleven and Koek, 1996[30]). Consequently, eye blinking behaviour has been applied as an indicator for cognitive states related to the dopaminergic system (Barbato et al., 2012[2]; Colzato et al., 2009[10]). Subjects that show higher eye blink rates preceding an experimental session are more attentive in a subsequent cognitive task (Colzato et al., 2008[9]) or demonstrate increased cognitive flexibility (Dreisbach et al., 2005[14]). In the latter study, the relation of this effect to the dopaminergic system was even underlined by genetic analyses. Chermahini and Hommel (2010[8]) challenged this finding by demonstrating an inverted U-shape-like relation between eye blink rate and cognitive flexibility. A medium eye blink rate appears to be best suited for high cognitive flexibility. On the other hand, convergent thinking was, in accordance with those previous findings, negatively related to eye blink rate.

Blink rate, along with blink duration, also changes during task endurance. For rather less demanding vigilance performance, blink rate and duration increases with time on task and emerging fatigue (McIntire et al., 2014[33]; McKinley et al., 2011[34]). This appears contradictory when the relation between eye blink rate and dopaminergic activity is considered since mental fatigue and decreasing motivation are related to a decrease in dopaminergic activity (Boksem and Tops, 2008[6]). However, it is possible that cognitive factors overwrite the relation between blink rate and dopamine during task performance. Blink rate decreases with higher cognitive and/or visual demand of tasks (Benedetto et al., 2011[5]; Fairclough et al., 2005[18]; Recarte et al., 2008[41]), and was found to be negatively correlated with task engagement (Fairclough and Venables, 2006[17]). In a visuo-motor tracking task, Drew (1951[15]) found that the adjustment of the blink rate to the difficulty of the task was not simply due to a global decrease in overall blink rate, but also by an alteration of blink timing. When difficulty of the task varied, participants performed blinks just before and just after periods of maximum difficulty. During maximum difficulty, blinks were completely inhibited.

In general, blink rate decreases when more attention is required to perform a task (Oh et al., 2012[38]). This is also underlined by the fact that blink rate increases with accumulating habituation to a certain task (Bonfiglio et al., 2011[7]). Additionally, the pharmacologically modulated level of dopamine by administration of a D2-antagonist and a D2-agonist was not significantly affecting blink rate when participants were looking at a virtual aquarium scene (van der Post et al., 2004[50]). Here, eye blink rates were apparently rather determined by the “task” than by the dopaminergic state. Similar effects can be observed when eye blinks were analysed during performance of cognitively demanding experiments. Performing in a Stroop task (Stroop, 1935[47]), participants blinked preferably around the execution of the response (Oh et al., 2012[38]). Also, in a card evaluation task, blinks were suppressed during presentation and evaluation of the visual material, and occur after a decision has been made (Fukuda, 2001[20]). Most interestingly, in the latter study, the number of blinks was increased when a card was relevant, compared to irrelevant cards. In semantic priming experiments, first blink latencies are reduced when stimulus pairs were semantically related and highly probable (Ichikawa and Ohira, 2004[25]). 

All these findings indicate that blinks may be related to visual information processing. It appears plausible that blinks are not executed as long as information has to be processed. In line with this notion, several studies report blinks to occur preferably at discrete breaks of the information flow, and more frequently during periods when little information is given. Blinks were frequently found at the end of a sentence while reading (Orchard and Stern, 1991[39]), after a decision (Fukuda, 1994, 2001[20]), during saccades (Fogarty and Stern, 1989[19]), or during a scene change while watching a movie (Nakano et al., 2009[36]). Furthermore, blinks are often found immediately after a manual response (Goldstein et al., 1992[21]; Oh et al., 2012[38]; Pivik and Dykman, 2004[40]; Siegle et al., 2008[42]). Blinks at the right time even seem to be able to enhance cognitive control for upcoming tasks, in terms of suppressing a dominant but interfering response (Verguts and Notebaert, 2009[51]). Performance was found to be enhanced after blinks compared to trials with no blink preceding (van Bochove et al., 2013[49]). Assuming that a blink indicates the closure of information processing of a given trial, more cognitive control capacity should be available for the following trial. 

As blinking interrupts visual perception for about 250 ms (Kennard and Glaser, 1964[29]), it is plausible to time blinks whenever such an interruption is less disturbing, and when the risk to miss important information is minimized (Baumstimler and Parrot, 1971[4]; Stern and Skelly, 1984[45]). This is not only because the eyelids are simply blocking visual input, but are also affected by a neural mechanism inhibiting processing of visual information (Volkmann et al., 1980[53]; Volkmann et al., 1982[52]). Remarkably, blinks are also inhibited and timed as described above, when no visual, but auditory stimuli are presented (Bauer et al., 1985[3]; Goldstein et al., 1985[22]). Thus, blinking is apparently inhibited during information processing regardless of stimulus modality. 

In summary, there is converging evidence that blinks are inhibited and executed at meaningful moments during cognitive and/or perceptual processing. Blinks are probably useful markers for the closure of such processes and may therefore provide information about processing time similar to manual responses. However, also other blink initiating mechanisms have been discussed. During finger tapping, eye blinks get synchronized with manual behaviour, indicating a possible overlap in the motor control of hand and eye movement systems (Cong et al., 2010[11]). 

Thus, the meaning and the functional relevance of eye blinks in experimental settings are not really uncovered so far. In the present study blinking behaviour was examined in three well-established cognitive experiments. Two experiments were long-term experiments that additionally allowed to address time on task effects that have been repeatedly reported for blinking behavior. Experiment 1 tested extended execution of a Simon task (Simon and Rudell, 1967[44]) that is characterized by a spatial stimulus-response conflict. Here, the shape of a stimulus defined the response side, while the stimulus location had to be ignored. Cognitive control, in terms of suppressing a dominant but interfering response was demanded for correct response selection. Perceptual demand was low, as there were only two kinds of easy to perceive and distinguishable stimuli presented one at a time. In contrast, Experiment 2 was a Change Detection task that included perceptual conflicts (Wascher and Beste, 2010[54]). Here, a luminance change had to be detected, while distracting changes of stimulus orientation had to be ignored. Response selection demanded less cognitive control, as the target position always gave response side unequivocally. Perceptual demand, on the other hand, was high, as the target stimulus' luminance change was difficult to perceive, especially when a salient distractor had to be ignored. Finally, data from a Continuous performance task - AX version (AX-CPT) were analyzed. In this task, participants had to respond to any 'X' that followed an 'A' as a cue. When a 'Y' followed the 'A' the prepared response had to be inhibited. Any 'X' or 'Y' following a 'B' as a cue were no-go stimuli not preceded by response preparation. Taken these three tasks together, it should be possible to distinguish between aspects of response conflicts, perceptual conflicts, as well as action control upon blinking behavior. 

## Methods

### Participants

15 healthy participants (10 female, 5 male, mean age = 24.5 yrs., sd = 3.56 yrs.) took part in Experiment 1, 11 healthy participants (8 female, 3 male, mean age = 25.25 yrs., sd = 2.73 yrs.) in Experiment 2, and 17 in Experiment 3 (10 female, 7 male, mean age = 25.1 yrs., sd = 3.16 yrs.). Participants had normal or corrected to normal vision and gave informed written consent to the experiment. All received either course credit or 10 € per hour for participation The study conformed to the Code of Ethics of the World Medical Association (Declaration of Helsinki) and was approved by the local Ethical Committee of the Leibniz Research Centre for Working Environment and Human Factors, Dortmund, Germany.

### Procedure and materials

All experiments took place in an electrically shielded, soundproof and dimly lit chamber. Participants were seated comfortably on a chair with each index finger loosely attached to the left and right response mechanism. Visual stimuli were presented by a VSG 2/5 graphic accelerator on a 22-inch CRT monitor (100 Hz) in a viewing distance of 120 to 140 cm, depending on the individual head position. 

### Experiment 1: Simon task

In experiment 1, participants had to respond to a square by pressing the right button, and to a diamond by pressing the left button (for details, see Möckel et al., 2015[35]). Both symbols could occur on either side, left or right from a fixation cross. Thus, the stimulus location could be either corresponding or non-corresponding to the response side. 

Each participant had to perform 3 blocks, consisting of 3 subblocks each. A subblock consisted of 480 trials. Blocks were separated by 5-10 minutes breaks. Stimuli were presented for 150 ms with a mean inter-trial-interval of about 2700 ms. Each subblock took about 20 minutes. Overall, the experiment lasted for 3 hours. 

### Experiment 2: Change Detection task

Experiment 2 was a Change Detection task (Wascher and Beste, 2010[54]), in which a luminance change had to be detected, while distracting changes of stimulus orientation had to be ignored. 

Participants were presented two frames of two laterally presented bars on either side of a fixation cross. Bars could be of high or low luminance (constant contrast to the mid-grey background), and were vertically or horizontally orientated. Two different pairs of bars were presented with a blank screen in between. Between the two stimulus frames the following features could change: 

(a) a luminance change of one bar (LUM), 

(b) a luminance and an orientation change of one bar (LOU = luminance-orientation unilateral), 

(c) a change in luminance of one and an orientation change of the other bar (LOB = luminance - orientation bilateral), or 

(d) an orientation change only (ORI). Participant had to press a button at the side where the luminance changed, while ignoring any other change. If no luminance change occurred this was a no-go trial (ORI). 

Participants had to perform 8 blocks of 384 trials each, including 96 no-go trials. The first and second pairs of bars were each presented for 70 ms with a 50-ms blank screen in between. After the fourth block, participants were given a short break of 5 minutes.

### Experiment 3: AX-CPT task

A paced sequence of four letters (A, B, X, Y) was presented in experiment 3. With a constant inter-stimulus-interval of 2000 ms, one of these letters was presented in the center of the screen. Within the sequence, letters were grouped in a way that any “A” or “B” was followed by an “X” or “Y”. The task of the participants was to press a button whenever an “X” followed an “A”. Any other letter was a no-go stimulus. The combination A-X was presented in 40 % of all stimulus pairs. A-Y was presented in only 10 %. The occurrence of B-X (10 %) and B-Y (40 %) was selected in a way that any letter was presented with the same frequency. In this task, any “A” denotes a cue for an upcoming target. A following “X” is a go-signal, whereas a “Y” after an “A” means that a prepared response has to be inhibited. Any “B” means that the next stimulus will not require an action, no matter whether this is an “X” or a “Y”.

### Recording and data processing

Vertical electro-oculograms (vEOGs) were recorded from two electrodes placed above and below the right eye. Bipolar vEOG was calculated for further analyses. Recordings in experiments 1 and 2 were processed by a 'BrainAmp MR plus' EEG amplifier (BrainProducts, Gilching, Germany) and digitised at 1000 Hz. Data was recorded with a Low Cutoff at DC and a High Cutoff at 250 Hz. For experiment 3, a NeurOne amplifier (Mega Electronic Ltd, Kuopio, Finland) was used with the same settings during recording as described above.

Manual responses were collected by custom-made force sensitive keys affixed to the armrests of the chair. Response force was analogously fed into the EEG system and sampled with the same frequency as the EOG signal. Responses were defined as every press on the force key that exceeded 200 cN. Correct responses were defined as key presses only on the correct key between 150 and 1500 ms after stimulus presentation for choice responses (Experiment 1 and Experiment 2), and between 100 and 1000 ms for go/no-go responses (Experiment 3). 

### Blink detection

For detection of blinks, local maxima were searched in the bipolar vEOG channel. In a first run (of a 2-step procedure) peaks in a moving 400 ms window were marked. Peaks were signed as possible blinks, if the difference between the peak amplitude and the lowest amplitudes 200 ms before and 200 ms after the peak were each larger than 100 µV, and when the difference between the peak value and the mean of the two lowest amplitudes was smaller than a maximum of 1000 µV. If no blink was detected, the window was moved by 40 ms to repeat the procedure. Following detected blinks the window was moved to the end of the detected blink. Based on this first sketch, amplitudes of individual blinks were used to calculate blink criteria for each participant. Procedure was then repeated with adjusted criteria to mark blinks for further processing.

### Measures

Response times and accuracy are reported as reference measures. Proportion of blinks in trials and first blink latencies (in correctly responded trials) were extracted based on the procedures described above. First blink latencies were selected as an indicator of task related blinking behavior. The difference between first blink latency and the related responses was defined as the response-blink latency. 

In experiment 1, response times, first blink latencies, and response-blink latencies were each entered into 3x3x2 ANOVAs with the factors block, subblock and correspondence (correspondent vs. non-correspondent stimulus and response location). In experiment 2, accuracy and proportion of blink in trials and first blink latencies were entered into 2x4x4 ANOVAs with the factors block, subblock and condition (4; LUM, ORI, LOU, LOB). Since ORI was a no-go condition, response times and response-blink latencies were entered into reduced ANOVAs (2x4x3), omitting ORI from the factor condition. In experiment 3, response times and response-blink latencies were not put into statistical analyses, because only in one condition (A-X) responses were executed. Accuracy, proportion of blinks in trials, and first blink latencies were analyzed separately for cues (“A”s and “B”s) and for targets (“X”s and “Y”s). The latter were classified depending on the preceding cue and entered into an ANOVA with the factors cue and target.

For factors with more than two levels, Greenhouse-Geisser adjusted p-values are reported where appropriate. If applicable, experimental conditions were contrasted via Tukey tests with Bonferroni adjusted p values. Additionally, effect sizes by means of partial eta squared (η_p_^2^) are reported for significant results. Signal analyses were performed on MATLAB®. All statistical analyses were conducted using GNU R (R Development Core Team, 2012). Plots were drawn using VEUSZ (Jeremy Sanders, 2013; http://home.gna.org/veusz/).

## Results

### Experiment 1

#### Manual behavior

Accuracy was higher when stimulus and response locations were corresponding, F(1,13) = 25.29, p < 0.001, η_p_^2^ = 0.66. Across blocks, no effect of time on task was found, F(1,13) = 0.07, p > 0.2. A decrease in performance within blocks slightly failed to reach significance, F(1,13) = 3.91, p = 0.070, η_p_^2^ = 0.23. The effect of correspondence increased with time on task, F(1,13) = 8.61, p = 0.012, η_p_^2^ = 0.40.

**Figure 1:**
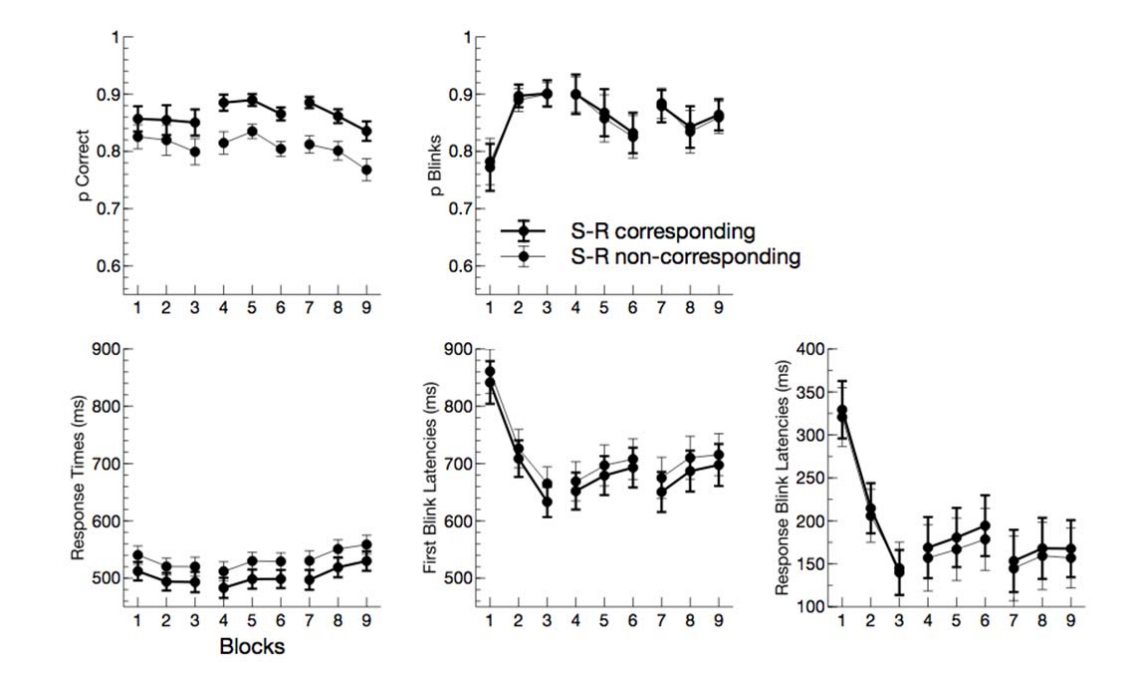
Behavioral and eye blink data in experiment 1. Accuracy and proportion of blinks in trials are depicted in the upper panel as a function of block (3 main blocks, consisting of 3 subblocks (1-3, 4-6, and 7-8). In the lower panel, response times, first blink latencies, and response-blink latencies are plotted (from left to right). Error bars are standard errors across participants.

Response times were prolonged for non-corresponding trials, F(1,13) = 36.76, p < 0.001, η_p_^2^ = .74. Additionally, an interaction of block by subblock, F(1,13) = 10.96, p = 0.006, η_p_^2^ = 0.46, indicated that response times decreased within the first block but increased within blocks thereafter. No other effect reached significance (all p > 0.05) (Figure 1(Fig. 1)).

#### Blink behavior

On average, in more than 85 % of all trials blinks were executed. Only the interaction of block by subblock reached significance for this measure, F(1,13) = 13.77, p = 0.003, η_p_^2^ = 0.51, indicating an increase of blinks in trials within the first block, whereas later blinks rather decreased within blocks. 

First blink latencies varied with S-R correspondence, F(1,13) = 16.76, p = 0.001, η_p_^2^ = 0.56. As for response times, the interaction of block by subblock, F(1,13) = 31.72, p < 0.001, η_p_^2^ = 0.71, describes an initial decrease of first blink latencies in the first block and a within-block increase for the blocks 2 and 3. 

Since this latter effect was more pronounced for first blink latencies compared to response times, also response-blink latencies showed the interaction of block by subblock, F(1,13) = 15.79, p = 0.002, η_p_^2^ = 0.55. Most interestingly, however, response blink latencies were reduced for non-corresponding trials, F(1,13) = 5.77, p = 0.032, η_p_^2^ = 0.31.

### Experiment 2

#### Manual behavior

Accuracy of manual responses varied across conditions, F(3,33) = 6.17, p = 0.002, η_p_^2^ = 0.36. Pairwise comparisons revealed that accuracy was reduced in conflict trials (LOB) compared to all other conditions (all ps < 0.001). No difference was found between the other conditions (all ps > 0.05). An additional interaction of block by subblock, F(1,11) = 13.84, p = 0.003, η_p_^2^ = 0.56, indicates that accuracy increased in the first block, but remained rather stable thereafter. 

**Figure 2:**
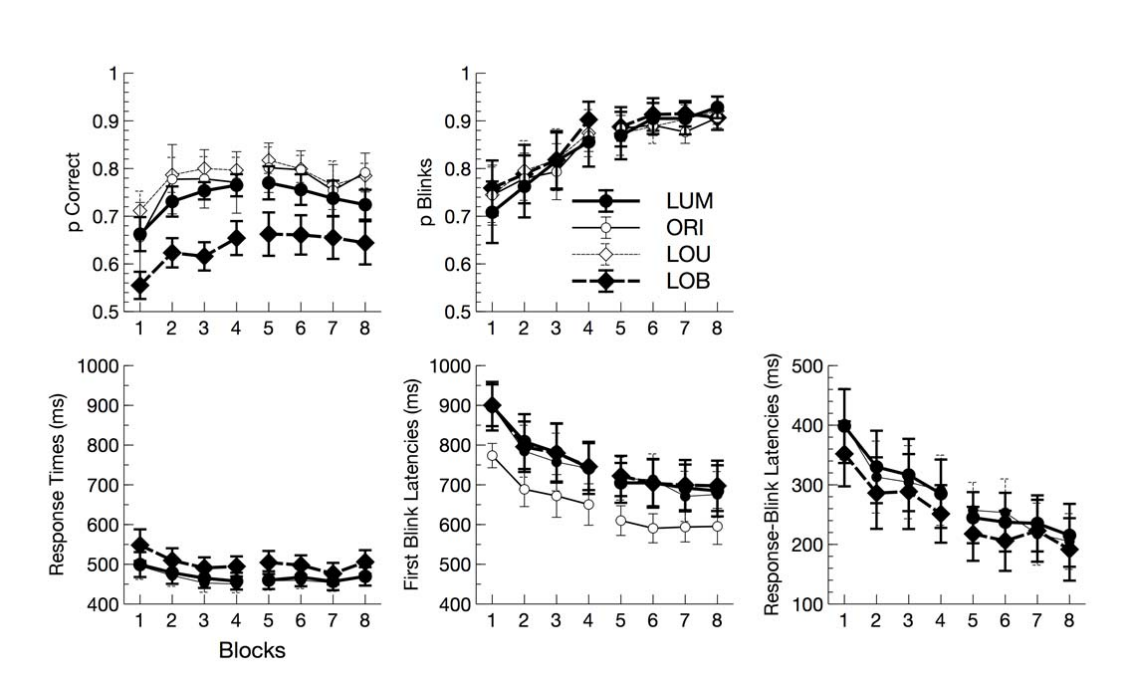
Behavioral and eye blink data in experiment 2. Accuracy and proportion of blinks for the different conditions are depicted in the upper panel as function of block (1-8). In the lower panel, response times, first blink latencies, and response-blink latencies are plotted (from left to right). Error bars are standard errors across participants.

For response times, only the main effect of condition reached significance, F(2,22) = 45.36, p < 0.001, η_p_^2^ = 0.80, with increased response times for the conflict condition compared to the other go conditions (all ps < 0.025), but no difference between LUM and LOU (ps > 0.05) (Figure 2(Fig. 2)).

#### Blink behavior

The percentage of trials in which blinks were executed was well above 85 % in all conditions. It increased across blocks, F(1,11) = 11.38, p = 0.006, η_p_^2^ = 0.51, and within blocks, F(1,11) = 9.84, p = 0.009, η_p_^2^ = 0.47. Additionally, the interaction of block by subblock, F(1,11) = 8.22, p = 0.015, η_p_^2^ = 0.43, indicated that the latter effect was stronger in the first block than in the second. 

In contrast to manual response times, first blink latencies varied with time on task. They decreased both across blocks, F(1,11) = 45.48, p < 0.001, η_p_^2^ = 0.81, as well as within blocks, F(1,11) = 13.94, p = 0.003, η_p_^2^ = 0.56. Also, the interaction of block by subblock reached significance, F(1,11) = 11.94, p = 0.005, η_p_^2^ = 0.52. The effect of condition on first blink latencies, F(3,33) = 10.36, p < 0.001, η_p_^2^ = 0.49, was due to reduced first blink latencies in the no-go condition (ORI) compared to all go conditions (all ps < 0.006). There was no difference between go conditions (all ps > 0.05). 

Response blink latencies mirrored the larger time on task effects in first blink latency compared to response times (Block: F(1,11) = 20.90, p = 0.001, η_p_^2^ = 0.66; Subblock: F(1,11) = 5.55, p = 0.038, η_p_^2^ = 0.34). Of key interest, however, was the effect of conditions upon this measure. Although overall an effect of conditions was observed, F(2,22) = 9.34, p = 0.001, η_p_^2^ = 0.46, none of the pairwise comparisons approached significance (all ps > 0.5).

### Experiment 3

#### Manual behavior

**Figure 3:**
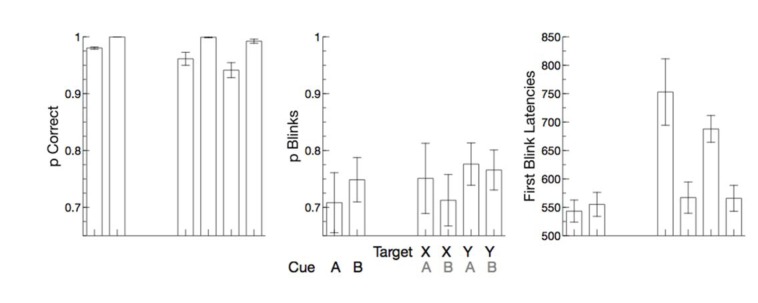
Behavioral and eye blink data in experiment 3. Accuracy, proportion of blinks, and First blink latencies are depicted for the different experimental conditions. Error bars are standard errors across participants.

Accuracy of manual responses was lower when a cue indicated a potentially upcoming response, F(1,11) = 111.21, p < 0.001, η_p_^2^ = 0.91. Also, when targets were presented, errors were committed predominantly after a response indicating cue, F(1,11) = 42.50, p < 0.001, η_p_^2^ = 0.79. No other effect reached significance (all ps > 0.5) (Figure 3(Fig. 3)).

#### Blink behavior

The proportion of blinks in trials did not vary, neither with the type of cue nor with the type of target presented (all ps > 0.05). First blink latency did not vary for the two different cues, but was increased when a target followed a response indicating cue, F(1,11) = 18.85, p = 0.001, η_p_^2^ = 0.63, but did not differ between go and no-go trials, F(1,11) = 2.53, p = 0.140, η_p_^2^ = 0.19.

## Summary

In summary, blink behavior varied systematically with experimental factors. Independently of the experimental setting, blinks were performed in the majority of trials. In both long-term experiments, first blink latency decreased and the proportion of blinks in trials increased with time on task. Despite this latter effect, blinks appear to be synchronized with manual responses. They appear at any moment of the experiment within a well-defined window following the button press. However, blinks were not simply entrained by the manual response (in sense of a common motor program), but occurred also in no-go trials. These no-go blinks showed the same time on task effects as blinks in go trials, indicating that blinking behavior was obviously related to stimulus processing, rather than to motor-related behavior (e.g., response preparation). In this sense, response conflicts but not perceptual conflicts affected response blink latencies. This effect was independent from time on task effects. That blink timing appears to be a marker of stimulus processing is supported by the observation that not the go/no-go distinction but response preparation (whether a probable response was indicated by the cue) affected blinking behavior in experiment 3. 

## Discussion

In the present study, we investigated the blinking behavior in three well-established cognitive tasks, measuring the resolution of response conflict (Experiment 1) and perceptual conflict (Experiment 2), as well as the control of prepared actions (Experiment 3). The proportion of blinks in trials as well as blink latencies and (where possible) response blink latencies were analyzed.

Assuming that eye blinks serve primarily the maintenance of the tear film on the eye, blinks should not occur more often than the average tear-break-up-time of 27 seconds (Sweeney et al., 2013[48]). With respect to the inter-trial intervals in the present experiments, about 5 to 10 % blinks in trials would be expected. This is far from the measured values, which ranged between 70 % and 90 % of trials in which a blink was executed. On average, every third second a blink was executed during the execution of a cognitive task. In the two long-term experiments (Experiments 1 and 2) the blink ratio increased within the first approximately 40 minutes of task execution and remained rather stable thereafter. This effect of time on task was accompanied by a variation of blink response times. The moment when a blink was executed during the execution of a cognitive task decreased strongly in the beginning of the experiment, and also reached a constant level. Both effects might be related to the functional meaning of blinks for information processing.

When considering eye blink behavior in cognitive tasks, one might assume that blinks might be inhibited as long as visual information is expected or occur at a time point that represents a meaningful moment in information processing (Stern and Skelly, 1984[45]; Stern et al., 1984[46]). Blink inhibition should avoid the missing of relevant signals (Holland and Tarlow, 1972[23], 1975[24]; Pivik and Dykman, 2004[40]). Such behavior should occur especially in the periods preceding stimulus presentation. Since the timing of stimulus presentation was widely predictable in the present experiments, this should have been an easy task. In fact, preceding stimulus presentation, we found an interval longer than 500 ms in which no blinks were executed. Such a pure “perceptual-based” blink inhibition should end with stimulus offset. In all experiments, however, blinks were suppressed along stimulus evaluation and response preparation. Blinks occurred never before the manual response (if applicable) was executed. After the initial phase (as described above), blinks occurred about 200 ms after the response in both long-term experiments. 

These data indicate that blinks denote a moment when all information processing in a given trial is finished (Bauer et al., 1985[3]; Fukuda, 1994[20]; Goldstein et al., 1985[22]; Holland and Tarlow, 1972[23], 1975[24]; Oh et al., 2012[37]; Orchard and Stern, 1991[39]; Siegle et al., 2008[42]; van Bochove et al., 2013[49]), and subjects prepare for the upcoming stimulus. Taken this explanation of blinking behavior, time on task effects on proportion of blinks and blink latencies might become clearer. Given the fact that at the beginning of any experiment, participants have to become familiar with the task, it is plausible that even after the response some post evaluation of the trial is done that delays the finalization of processing. This would explain the extension of blink latencies. With this behavior, the termination of cognitive processing in a given trial becomes temporally closer to the next potential stimulus. This might lead to increasing proportion of blink inhibition to avoid information loss (Nakano et al., 2009[36]), which would explain the reduced proportion of blinks in trials in the beginning of an experiment.

Most interestingly, however, both blinks in no-go trials and experimental effects on response-blink latencies indicated that it is not simply the manual response that initiates blinking behavior. It has been assumed that blinks might be entrained by manual response, because finger tapping blinks occur simultaneously with manual behavior (Cong et al., 2010[11]). However, in Experiments 2 and 3 blinks were also executed when the trial required no manual response. In Experiment 2, no-go blinks showed the same time on task effects as go blinks. In Experiment 3, blinks were delayed when a response had to be executed or inhibited, compared to trials in which a preceding cue had already indicated that the upcoming stimulus will be irrelevant. Thus, stimulus evaluation appears to play a major role for the timing of blink execution.

Specification of this assumption comes from the evaluation of other experimental effects on blink latencies. Response blink latencies were reduced when a response conflict had to be resolved, i.e., when a non-corresponding trial had to be processed compared to corresponding trials. Perceptual conflicts such as in Experiment 2 were not mirrored in response blink latencies. In the Simon task (Simon, 1969[43]) used in Experiment 1, it is assumed that the correspondence effect is (at least in parts) independent from target stimulus evaluation (De Jong et al., 1994[12]; Wascher et al., 2001[55]). Automatic response activations may affect response selection independently from semantic stimulus processing (see also Kornblum et al., 1990[31]). Thus, together with the outcome of Experiment 3, the most probable explanation of blinking behavior is that blinks are initiated when stimulus evaluation is complete. In this case, prolonged response selection would shorten the interval between response and blink. Since perceptual conflicts prolong stimulus evaluation, but not response selection, it becomes clear that in this case also response blink latencies remain unaffected.

In summary, when analyzing blinking behavior in cognitive tasks, it becomes evident that, at least in experimental situations, blinks are far from stochastically executed at a casual moment in time. Also, strategic inhibition of blinks to avoid information loss is only half the truth. Although the latter mechanism might have influenced the proportion of blinks in trials, the core trigger of blink execution appears to be the finalization of stimulus evaluation. Based on the findings presented here, blinks might provide a reliable marker of cognitive processing speed even in no-go situations. Moreover, assuming that blinks in natural situations follow the same principle, they might become useful measures for information segmentation also outside the laboratory. 

## Notes

Edmund Wascher and Holger Heppner share the first authorship.

## Conflict of interest

The authors declare no conflict of interest.
